# The feasibility of Chinese massage as an auxiliary way of replacing or reducing drugs in the clinical treatment of adult type 2 diabetes: A systematic review and meta-analysis

**DOI:** 10.1097/MD.0000000000021894

**Published:** 2020-08-21

**Authors:** Xiaolin Zhang, Di Cao, Minhui Yan, Mingjun Liu

**Affiliations:** a College of Acupuncture and Massage, Changchun University of Chinese Medicine; b Department of rehabilitation, Changchun Hospital of Chinese Medicine; c Acupuncture and Massage Center of the Third Affiliated Clinical Hospital of Changchun University of Chinese Medicine, Changchun, China.

**Keywords:** Chinese massage, drug reducing therapy, physiotherapy evidence database scale, systematic review, type 2 diabetes mellitus

## Abstract

**Background:**

At present, metformin is mainly used in the treatment of type 2 diabetes mellitus (T2DM). When the therapeutic effect is achieved, there are side effects and secondary failure will occur if taken for a long time. It is of great significance to actively explore the clinical scheme of reducing drug use while ensuring the therapeutic effect of T2DM.

**Objective:**

To evaluate the feasibility of Chinese massage (CM) in the treatment of T2DM.

**Methods:**

Literature retrieval is divided into 2 aspects: Electronic Retrieval and Personal Check. We will search PubMed, EMBASE, CNKI, Cochrane Central, which were registered in international clinical trials registry platform systems, select all eligible studies published before November 2, 2019, and use Personal Check method to retrieve papers, conference papers, ongoing experiments, internal reports, and so on. With fasting blood glucose, 2-hour fasting blood glucose, glycosylated hemoglobin, and insulin index as the main observation indexes, we also pay attention to traditional Chinese medicine syndrome score scale, insulin resisting index, body mass index , serum total cholesterol, Curative effect and the occurrence of all adverse reactions in drug treatment.

Of the research group 2 researchers respective selected literature, extracted data, and evaluated the risk of bias. After that we used Revman 5.7 and Stata 12.1 statistical software for meta-analysis.

**Results:**

A total of 769 subjects were included in 10 studies for meta-analysis. Compared with metformin hydrochloride tablets, CM plus baseline treatment can reduce fasting plasma glucose (weighted mean difference [WMD] = −0.33, 95% confidence interval [CI] [−0.54, −0.13], *Z* = 3.15, *P* = .002), 2 hours postprandial blood glucose (WMD = −0.52, 95% CI [−0.70, −0.34), *Z* = 5.66, *P* < .00001], hemoglobin A1c (WMD = 0.12, 95% CI [0.04, 0.20], *Z* = 2.94, *P* = .003), fasting insulin (WMD = −3.59, 95% CI [−5.56, −1.42], *Z* = 10.29,

*P* < .00001), traditional Chinese medicine syndrome score scale (WMD = −4.55, 95% CI [−7.58, −1.51], *Z* = 2.94, *P* = .003),

homeostasis model assessment of insulin resistance (WMD = −1.76, 95% CI [−2.25, −1.27), *Z* = 7.08, *P* < .00001),

body mass index (WMD = −1.28, 95% CI [−1.65, −0.92], *Z* = 6.91, *P* < .00001), serum total cholesterol (WMD = −1.01, 95% CI [−1.14, −0.83], *Z* = 15.51, *P* < .00001), meanwhile, the effective rate was increased (risk ratio [RR] = 1.31, 95% CI [1.21, 1.42], *Z* = 6.57, *P* < .00001).

**Conclusion:**

CM combined with metformin hydrochloride tablet has a synergistic effect. It can not only be used as an auxiliary treatment of T2DM, but also as an important reference way of reducing drug treatment of T2DM, improving Clinical Efficacy and reducing adverse reactions.

**Systematic review registration:**

PROSPERO CRD42020158839

## 1 Introduction

Diabetes is one of the most serious chronic non communicable diseases that threaten human health. According to the latest statistics of the International Diabetes Federation in 2013, the prevalence of diabetes in adults aged 20 to 79 is to the percentage of 8.3%, which has reached 382 million, and is on the rise. It is estimated that by 2035, there will be 592 million diabetics in the world,^[1]^ while the number of diabetics in China will reach to 143 million, of which 95% will be type 2 diabetes (T2DM).^[2]^

Diabetes has a great harm, and it is full of complexity, such as cerebral thrombosis, angina, myocardial infarction, neuropathy, retinopathy, foot ulceration, infection, can cause death and disability, which has become the sixth major disabling factor.^[3]^ At present, the main clinical treatment methods are sulfonylureas, differential elimination, metformin, glitaZone, glycosidase inhibitors, and so on. But there are also gastrointestinal reactions, anemia, red blood cell reduction, liver and kidney function damage, serious lactic acid poisoning, and so on. And after drug using for a long time will lead to secondary failure.^[4]^ Based on the current situation, it is of great significance to actively explore the clinical scheme of reducing drug use meanwhile not affecting the therapeutic effect of T2DM.

With high clinical effects, lower side effects, high acceptability, and adaptability in physical treatment method of Chinese massage (CM). It has been included in the standard of diagnosis and treatment of diabetes in traditional Chinese medicine.^[5]^ T2MD clinical treatment, combined with massage treatment on the basis of oral drugs, can not only enhance the curative effect, but also reduce the gastrointestinal discomfort caused by medicine. Massage can play a better complementary role.

Therefore, this review systematically evaluates the existing clinical research literature of traditional CM in the treatment of adult T2DM, and it also discusses the feasibility of its clinical drug reduction treatment, in order to provide reference for clinicians to reduce the treatment of T2DM.

## 2 Data and methods

### 2.1 Study Registration

This research plan has been registered in PROSPERO with the registration number of CRD42020158839, and the specific plan has been published on its official website (https://www.crd.york.ac.uk/prospero/display_record.php?RecordID=158839). This study is carried out in strict accordance with the preliminary design framework.

### 2.2 Literature search

By using the combination of subject words and free words, we systematically searched CNKI, PubMed, EMBASE, Cochrane Central, who international clinical trials registry platform, and collected the clinical randomized controlled trials (RCT) of CM assisted treatment of adult T2DM. The retrieval time was from the establishment of the database to November 2, 2019. English search words are “type 2 diabetes,” “type 2 diabetes mellitus,” “traditional Chinese medicine,” “randomiZed controlled,” and so on; Chinese search words are “type 2 diabetes,” “massage,” “massage,” “randomiZed controlled clinical experiment,” “randomiZed controlled clinical observation.” If necessary, contact the author of the paper for incomplete information.

### 2.3 Inclusion criteria for study selection

#### 2.3.1 Types of studies

Randomized controlled study (whether blind method or assignment concealment is adopted or not)

#### 2.3.2 Types of participants

According to the diagnostic criteria, the basic indicators of T2DM were clear baseline, age, course, number, and gender of patients were unlimited.

#### 2.3.3 Types of interventions

The experimental group was treated with CM combined with metformin hydrochloride tablets; the control group was treated with metformin hydrochloride tablets.

#### 2.3.4 Types of outcome measures

At least including fasting plasma glucose (FPG), fasting insulin (FINs), 2 hours postprandial blood glucose (2hPG), homeostasis model assessment of insulin resistance (HOMA-IR), serum total cholesterol (TC), triglyceride (TG), and any 2 items of effective rate [effective rate = (recovery + significant effect + effective)/total number of cases].

### 2.4 Literature exclusion criteria

Repeated published (only the earliest one is reta kept for multilingual publications);

Only abstract is published, and the full text of the literature cannot be obtained after contacting with the author;

Literature with incomplete data or obvious errors;

In the intervention group, CM combined with metformin hydrochloride tablet was not the main intervention literature;

Literature reported subjects unfinished treatment ≥20%.

### 2.5 Literature screening and data extraction

According to the PRISMA flow chart, 2 independent researchers Zhang Xiaolin and Yan Minghui searched, screened, extracted, and cross checked the literature respectively. Cao Di, the third researcher, worked together to resolve the divergent literature and ensure that the final consistency was no less than 80%. The extracted data include:

(1) Basic information in the study: the author, the years, the country or region, the sample size, the age, the course of disease, the gender, and so on;
(2) Baseline characteristics and intervention measures of the study;
(3) Information related to bias risk assessment;
(4) Information related to main and secondary outcome indicators.

### 2.6 Risk assessment of literature bias

The risk of literature bias was assessed using a third-party physical therapy evidence database (sponsored by the Australian Institute of Neuroscience [neura]). If the platform cannot be evaluated, Zhang Xiaolin and Yan Minghui will evaluate it according to the bias risk assessment tool provided by Cochrane Handbook 5.2.0, and generate the bias risk map by Revman 5.3 software.

### 2.7 Statistical methods

We Used Revman 5.3 software provided by Cochrane Collaboration Network to make flow chart and prism scale. The meta-analysis was carried out by Using Stata 12.0 software. Weighted mean difference (WMD) and its 95% confidence interval (CI) are used as statistical measures for effect analysis; *Q*-test and *I*^2^ are used to evaluate heterogeneity. If *I*^2^ ≥ 50% or *P* < .05, heterogeneity can exist in the results of each study. Random effect model is used for combination analysis, Galbraith map is drawn to detect the source of heterogeneity; Egger test is used to detect whether publication bias exists; and subgroup analysis is used to compare the effect of different factors.

## 3 Results

### 3.1 Literature retrieval process and results

**Figure 1:**
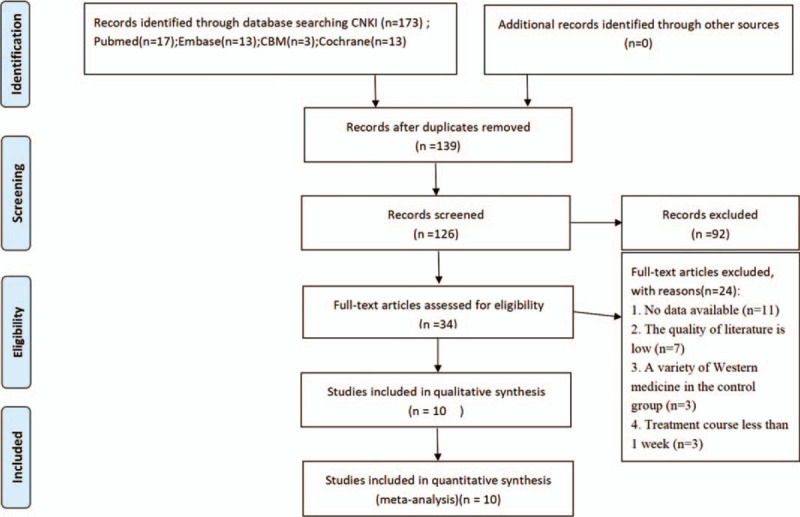
PRISMA flow diagram of study and exclusion.

A total of 219 literature were retrieved, including 173 in Chinese and 46 in English. 139 literature were screened after duplicating check, 92 were removed after reading the title/abstract, 34 were left for full-text reading, 24 were removed after reading the full text, and finally 10 documents were included for qualitative analysis, and 10 were included in meta-analysis. The process of literature search and selection was shown in Figure 1.

### 3.2 Basic characteristics of the study

A total of 10 studies were included, published from 2008 to 2019, including 4 from Jilin,^[11–14]^ 2 from Guangdong,^[8,9]^ 2 from Jiangsu,^[7,15]^ 1 from Beijing,^[6]^ 1 from Heilongjiang,^[10]^ all of which were RCTs published in the form of Chinese literature. All patients were adults with T2DM. 392 cases in the intervention group and 377 cases in the control group. All the studies reported the general data of patients’ age, gender, course of disease in the form of text or chart, and the difference was not statistically significant. The basic characteristics of the study were shown in Table 1.

**Table 1:**
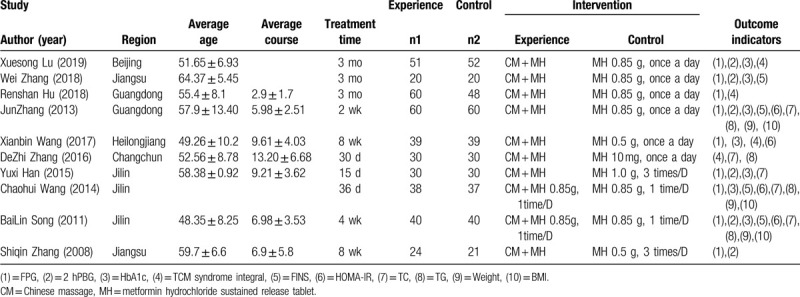
Meta-analysis of the feasibility of reducing drugs in massage treatment of type 2 diabetes.

### 3.3 Assessment on included study of risk of bias

**Figure 2:**
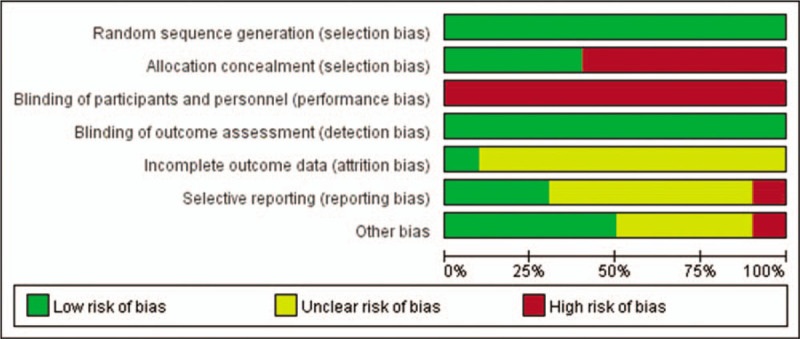
Risk chart of literature deviation for the feasibility of CM assisted drug reduction in adult T2DM mellitus patients. CM = Chinese massage, T2DM = type 2 diabetes mellitus.

The risk of literature bias was assessed used a third-party physical therapy evidence database (sponsored by the Australian Institute of Neuroscience [neura]), as shown in Table 2 below. As in Figure 2 for the proportion of risk of literature quality bias in Cochrane Handbook 5.20.

**Table 2:**
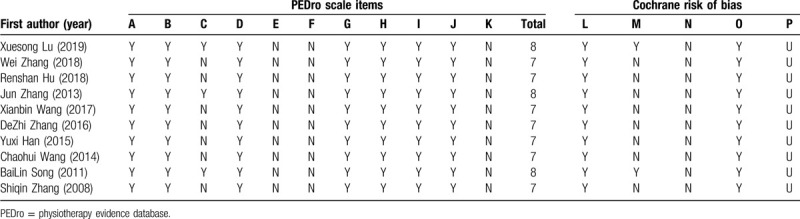
Quality assessment included in the study.

### 3.4 Meta-analysis results

#### 3.4.1 Main outcome indicators

##### 3.4.1.1 Main outcome indicators - FPG

**Figure 3:**
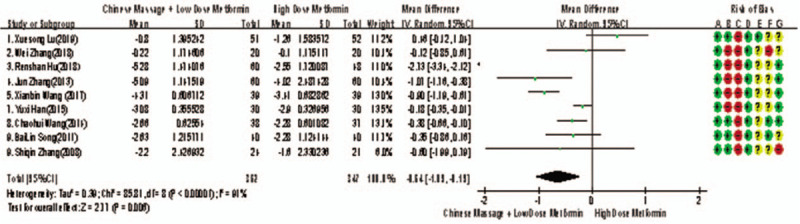
The meta-analysis forest chart of the feasibility of improving FPG by reducing drug treatment in adult T2DM patients assisted by CM. CM = Chinese massage, FPG = fasting plasma glucose, T2DM = type 2 diabetes mellitus.

**Figure 4:**
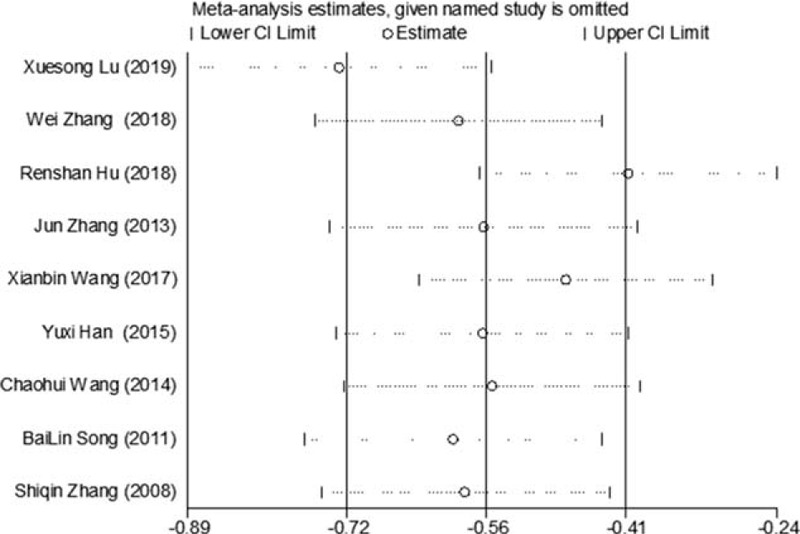
Sensitivity analysis of the feasibility of improving FPG by CM assisted with drug reduction in adult patients with T2DM. CM = Chinese massage, FPG = fasting plasma glucose, T2DM = type 2 diabetes mellitus.

**Figure 5:**
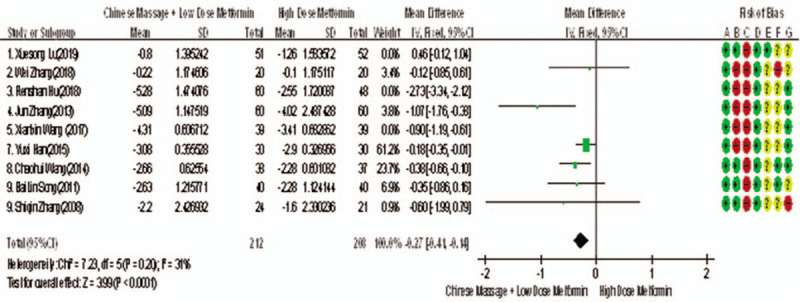
The forest map of meta-analysis on the feasibility of improving FPG removal by reducing drug treatment in adult T2DM patients assisted by CM. CM = Chinese massage, FPG = fasting plasma glucose, T2DM = type 2 diabetes mellitus.

Of the 10 included studies, 9 reported changes in FPG before and after treatment,^[6–11,13–15]^ a total of 709 subjects. There was heterogeneity between the 2 groups (*P* < .00001, *I*^2^ = 91%). A meta-analysis was carried out by using the random effect model. The results showed that there was statistically significant difference in FPG between the 2 groups after treatment (WMD = −0.64, 95% CI [−1.09, −0.19], *Z* = 2.77, *P* = .0006), As in Figure 3. The sensitivity analysis was shown in Figure 4. It was found that there is a risk of deviation in the study of Lu Xuesong (2019), Hu Renshan (2018), and Wang Xianbin (2017). When these 3 studies are excluded, the heterogeneity between the studies decreases (*P* = .20, *I*^2^ = 31%). The source of heterogeneity may be that the intervention time of the 3 studies is different, the other 6 groups are similar, and the results are shown in Figure 5. The difference of FPG index between the 2 groups after treatment is statistically significant (WMD = −0.33, 95% CI [−0.54, −0.13], *Z* = 3.15, *P* = .002). The results showed that the improvement of FPG by CM combined with metformin hydrochloride tablets was more significant than that by metformin hydrochloride tablets, which indicated that massage can be used as a clinical drug reducing therapy for T2MD to improve FPG.

##### 3.4.1.2 Main outcome indicators - 2hPG

**Figure 6:**
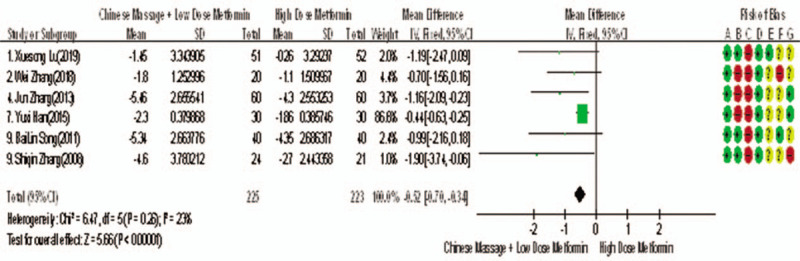
Meta-analysis forest chart of the feasibility of CM to assist the adult patients with T2DM mellitus to improve 2 hPG. 2hPG = 2 hours postprandial blood glucose, CM = Chinese massage, T2DM = type 2 diabetes mellitus.

Of the 10 included studies, 6 reported changes before and after 2hPG treatment,^[6,7,9,14,15]^ a total of 448 subjects. The homogeneity between the 2 groups was good (*P* = .26, *I*^2^ = 23%). The fixed effect model was used for meta-analysis. The results showed that there was significant difference between the 2 groups in 2 hours after treatment (WMD = −0.52, 95% CI [−0.70, −0.34), *Z* = 5.66, *P* < .00001), As in Figure 6. The results showed that the effect of massage combined with metformin hydrochloride tablets on 2hPG was more significant than that of metformin hydrochloride tablets, indicating that massage can be used as a clinical medicine reducing therapy for T2MD to improve 2 hPG.

##### 3.4.1.3 Main outcome indicator HbA1c

**Figure 7:**
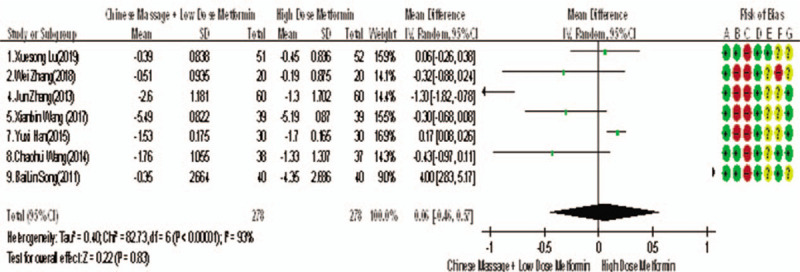
The forest map of meta-analysis on the feasibility of improving HbA1c with CM and reducing drug treatment in adult type 2 diabetic patients. CM = Chinese massage, HbA1c = hemoglobin A1c.

**Figure 8:**
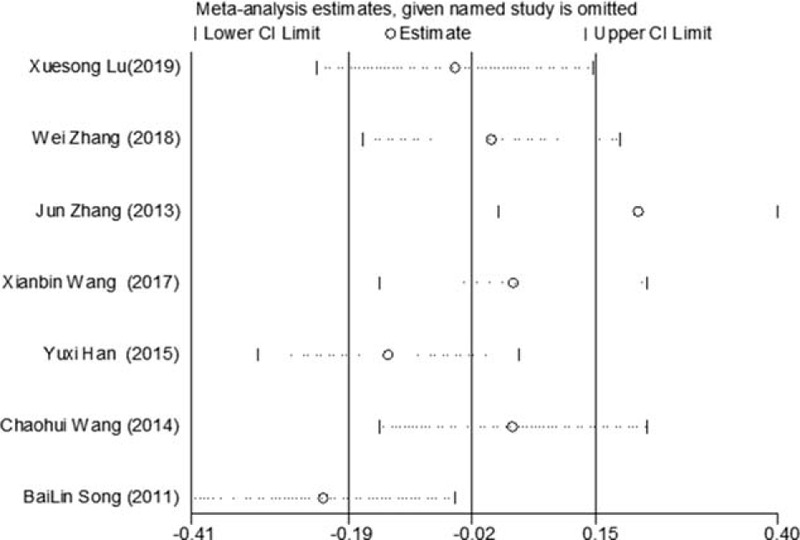
Sensitivity analysis of the feasibility of reducing drug treatment to improve HbA1c in adults with T2DM mellitus assisted by CM. CM = Chinese massage, HbA1c = hemoglobin A1c, T2DM = type 2 diabetes mellitus.

**Figure 9:**
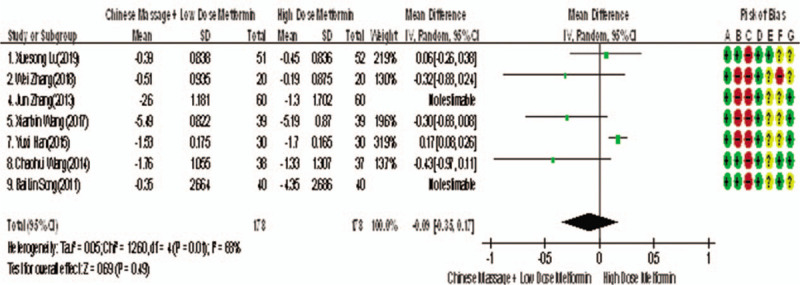
The meta-analysis forest chart of the feasibility of improving HbA1c after elimination by CM assisted with reducing drug treatment in adult patients with T2DM. CM = Chinese massage, HbA1c = hemoglobin A1c, T2DM = type 2 diabetes mellitus.

Of the 10 studies mentioned, 7 reported changes in HbA1c before and after treatment,^[6,7,9,10,12–14]^ a total of 556 subjects. There was heterogeneity between the 2 groups (*P* < .00001, *I*^2^ = 93%). There was no significant difference in HbA1c between the 2 groups after treatment by meta-analysis using random effect model (WMD = −0.06, 95% CI [−0.46, −0.57], *Z* = 0.22, *P* = .83), as shown in Figure 7. The sensitivity analysis was shown in Figure 8, and it was found that there is a risk of deviation in Zhang Jun (2013) and song Bolin (2011). When the 2 studies are excluded, the heterogeneity between the 2 studies decreases (*P* = .01, *I*^2^ = 68%). The origin of heterogeneity may be that the duration of the 2 study intervention subjects is longer, and the other 5 groups are relatively short, and the results are shown in Figure 9. The HbA1c difference between the 2 groups after treatment is statistically significant (WMD = 0.12, 95% CI [0.04, 0.20], *Z* = 2.94, *P* = .003). The results show that CM combined with metformin hydrochloride tablets can improve HbA1c more significantly than metformin hydrochloride tablets, indicating that massage can be used as a clinical medicine reducing treatment for T2MD to improve HbA1c.

##### 3.4.1.4 Main outcome indicators - FINs

**Figure 10:**
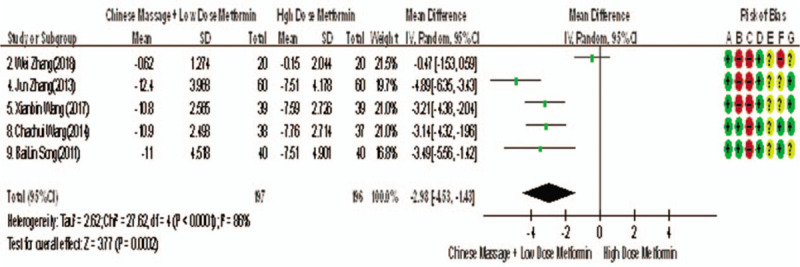
The forest map of meta-analysis on the feasibility of improving FINs by CM in the treatment of reducing drugs for adult T2DM. CM = Chinese massage, FINs = fasting insulin, T2DM = type 2 diabetes mellitus.

**Figure 11:**
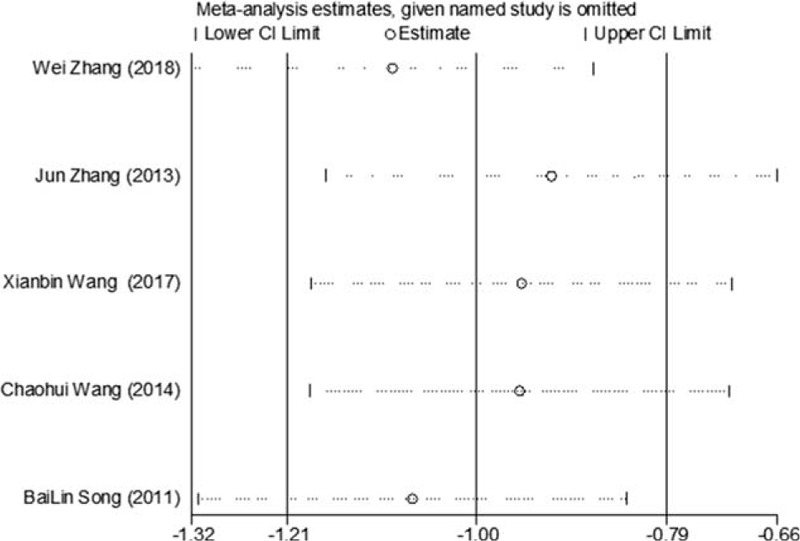
Sensitivity analysis of the feasibility of traditional CM assisted with reducing drug treatment to improve FINs in adults with T2DM mellitus. CM = Chinese massage, FINs = fasting insulin, T2DM = type 2 diabetes mellitus.

**Figure 12:**
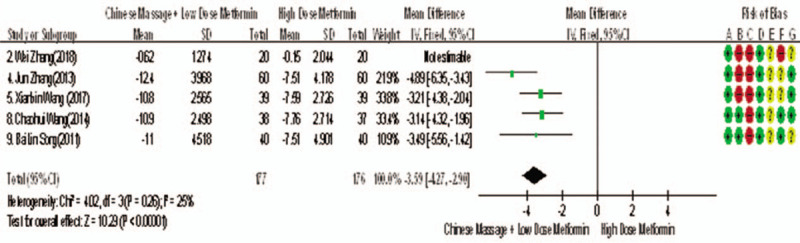
The forest map of meta-analysis on the feasibility of improving FINs after removal by CM in the treatment of T2MD. CM = Chinese massage, FINs = fasting insulin, T2DM = type 2 diabetes mellitus.

Of the 10 studies included, 5 reported changes in FINs before and after treatment,^[7,9,10,13,14]^ a total of 393 subjects. There was heterogeneity between the 2 groups (*P* < .00001, *I*^2^ = 86%). The results clearly showed that there was no significant difference in FINs between the 2 groups (WMD = −3.94, 95% CI [−5.56, −1.42], *Z* = 9.12, *P* < .00001), As in Figure 10. The sensitivity analysis was shown in Figure 11. We can found that study Zhang Wei (2018) has a risk of deviation. When this study is excluded, the heterogeneity between the studies decreases (*P* = .26, *I*^2^ = 25%). The origin of heterogeneity may be that the average age of the subjects involved in this study is relatively high, and the other 4 groups are relatively low, and the results indicated that there is a statistically significant difference in FINs index between the 2 groups after treatment (WMD = −3.59, 95% CI [−5.56, −1.42], *Z* = 10.29, *P* < .00001), see Figure 12. The results shows that CM combined with metformin hydrochloride tablets improved FINs more significantly than metformin hydrochloride tablets. The fact is that massage can be used as a clinical medicine reducing therapy to improve FINs in T2MD.

#### 3.4.2 Secondary outcome indicators

##### 3.4.2.1 Secondary outcome measures - effectiveness

**Figure 13:**
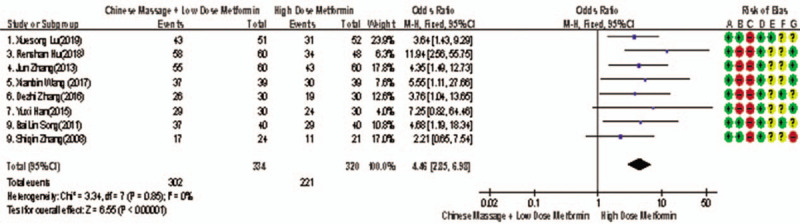
Meta-analysis forest chart of the feasibility of improving the efficiency of CM assisted treatment of T2MD in adults. CM = Chinese massage, T2DM = type 2 diabetes mellitus.

Of the 10 studies mentioned, 8 reported the overall improvement efficiency of T2MD,^[6,8–12,14,15]^ a total of 654 subjects. The homogeneity between the 2 groups was good (*P* = .97, *I*^2^ = 0%). The fixed effect model was used for meta-analysis. The results shows that there was statistically significant difference in FINs between the 2 groups after treatment (RR = 1.31, 95% CI [1.21, 1.42], *Z* = 6.57, *P* < .00001), as shown in Figure 13. The results indicates that the effect of CM combined with metformin hydrochloride tablets on the improvement of FINs was more valuable than that of metformin hydrochloride tablets, manifesting that massage can be used as an effective means of clinical drug reduction treatment of T2MD.

##### 3.4.2.2 Secondary outcome index - traditional Chinese medicine syndrome score scale (TCMSSS)

**Figure 14:**
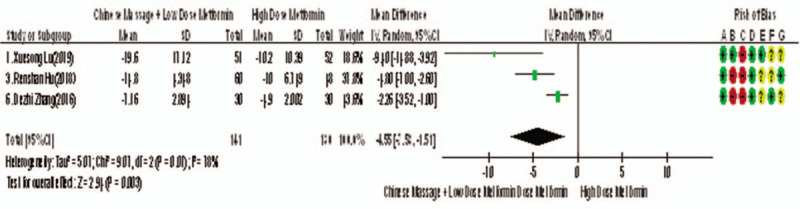
Meta-analysis forest chart of the feasibility of CM assisted with the reduction of TCM treatment for the improvement of TCMSSS in adult type 2 diabetic patients. CM = Chinese massage, TCMSSS = traditional Chinese medicine syndrome score scale.

Among the 10 studies, 3 reported the changes of TCMSSS before and after treatment,^[6,9,11]^ 271 subjects in total. There was heterogeneity between the studies (*P* = .01, *I*^2^ = 78%), and meta-analysis was carried out by using the random effect model. The results manifesting that there was statistically significant difference between the 2 groups in the integration of TCM syndromes after treatment (WMD = −4.55, 95% CI [−7.58, −1.51], *Z* = 2.94, *P* = .003), as shown in Figure 14. It shows that the improvement of CM combined with metformin hydrochloride tablets is more significant than that of metformin hydrochloride tablets, indicating that massage can be used as clinical medicine reducing therapy to improve the integration of TCM syndromes in T2MD.

##### 3.4.2.3 Secondary outcome indicator HOMA-IR

**Figure 15:**
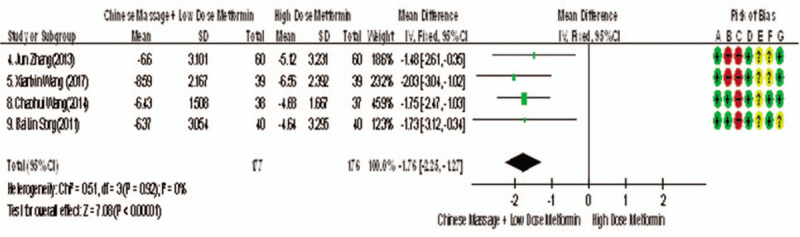
The meta-analysis forest chart of the feasibility of CM assisted with the reduction of TCM treatment and improvement of TCMSSS in adult T2MD patients. CM = Chinese massage, TCMSSS = traditional Chinese medicine syndrome score scale, T2DM = type 2 diabetes mellitus.

Of the 10 studies included, 4 reported changes before and after HOMA-IR treatment,^[9,10,13,14]^ a total of 353 subjects. The homogeneity of each study was good (*P* = .92, *I*^2^ = 0%). The fixed effect model was used for meta-analysis. The results showed that there were statistically significant differences in HOMA-IR between the 2 groups after treatment (WMD = −1.76, 95% CI [−2.25, −1.27], *Z* = 7.08, *P* < .00001], as shown in Figure 15. The results showed that CM combined with metformin hydrochloride tablets improved HOMA-IR more significantly than metformin hydrochloride tablets, indicating that massage can be used as a clinical medicine reducing treatment for T2MD to improve HOMA-IR.

##### 3.4.2.4 Secondary outcome measure BMI

**Figure 16:**
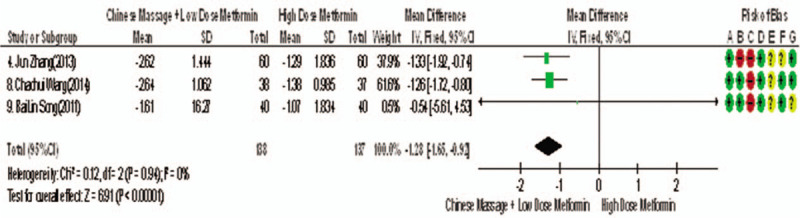
Meta-analysis forest chart of the feasibility of CM assisted with reducing drug treatment to improve BMI index in adult T2MD patients. BMI = body mass index, CM = Chinese massage, T2DM = type 2 diabetes mellitus.

Among the 10 studies, 3 reported changes in BMI before and after treatment^[9,13,14]^, a total of 275 subjects. The homogeneity between the studies was good (*P* = .94, *I*^2^ = 0%), and the fixed effect model was used for meta-analysis. The results showed that there was statistical significance in BMI index between the 2 groups after treatment (WMD = −1.28, 95% CI [−1.65, −0.92], *Z* = 6.91, *P* < .00001], as shown in Figure 16. The results showed that the improvement of BMI index of CM combined with metformin hydrochloride tablets was more significant than that of metformin hydrochloride tablets, indicating that massage can be used as a clinical drug reduction therapy to improve BMI index of T2MD.

##### 3.4.2.5 Secondary outcome measure TC

**Figure 17:**
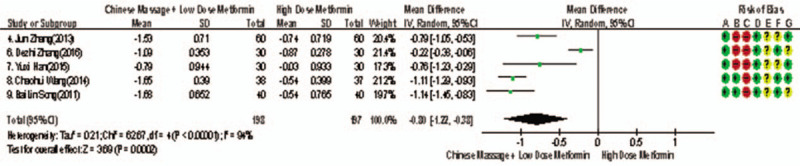
The meta-analysis forest chart of the feasibility of CM assisted with reducing drug treatment to improve TC in adults with T2MD. CM = Chinese massage, T2DM = type 2 diabetes mellitus.

**Figure 18:**
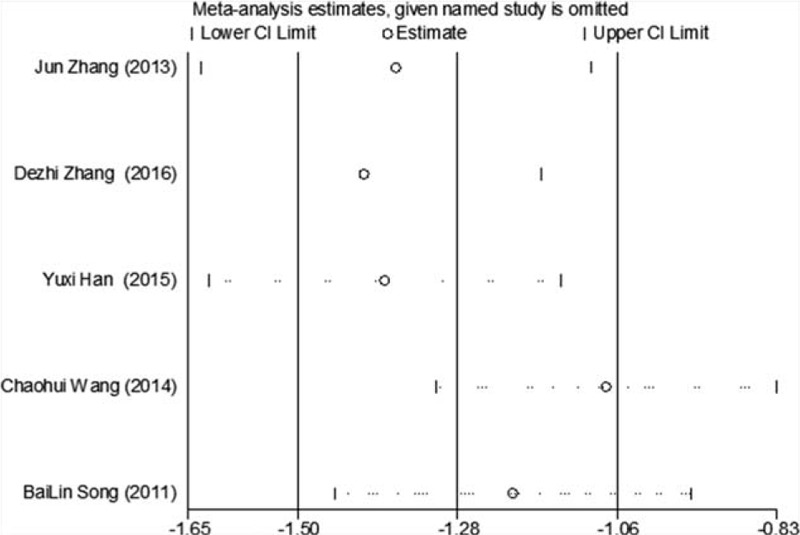
Sensitivity analysis of the feasibility of traditional Chinese medicine massage assisted with reducing drug treatment to improve FINs in adults with T2MD. FINs = fasting insulin, T2DM = type 2 diabetes mellitus.

**Figure 19:**
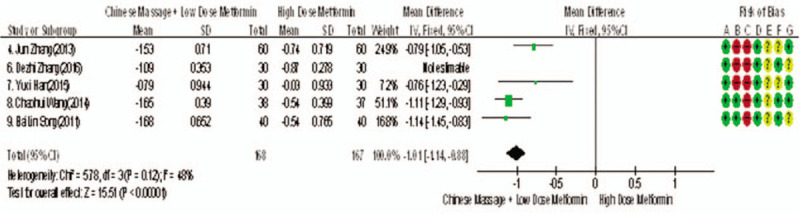
Meta-analysis forest chart of the feasibility of CM assisted with reducing drug treatment to improve TC in adults with T2MD. CM = Chinese massage, T2DM = type 2 diabetes mellitus.

Of the 10 studies included, 5 reported changes in TC before and after treatment,^[9–15]^ a total of 395 subjects. There was heterogeneity between the 2 groups (*P* < .00001, *I*^2^ = 94%). The results showed that there was no significant difference in TC between the 2 groups (WMD = −0.80, 95% CI [−1.22, −0.38], *Z* = 3.69, *P* = .0002), see Figure 17. The sensitivity analysis is shown in Figure 18. It is found that there is a risk of deviation in study Zhang DeZhi (2016). When this study is excluded, the heterogeneity between the studies decreases (*P* = .12, *I*^2^ = 48%). The source of heterogeneity may be that the average duration of this study intervention object is longer, and the other 4 groups are similar and relatively short. The results show that there is a statistically significant difference in TC index between the 2 groups after treatment (WMD = −1.01, 95% CI [−1.14, −0.83], *Z* = 15.51, *P* < .00001), see Figure 19. It shows that CM combined with metformin hydrochloride tablets can improve TC more significantly than metformin hydrochloride tablets, indicating that massage can be used as a clinical medicine reducing treatment for T2MD to improve TC.

### 3.5 Analysis of negative reactions of CM combined with western medicine in T2MD

Negative events were mentioned in 10 studies, and a small number of decreased appetite and nausea were mentioned in the control group; and another small number of decreased appetite and nausea were mentioned in 3 study groups, also there was no difference between the 2 groups. Follow up treatment of negative reactions was not mentioned in all studies.

### 3.6 Publication bias analysis

**Figure 20:**
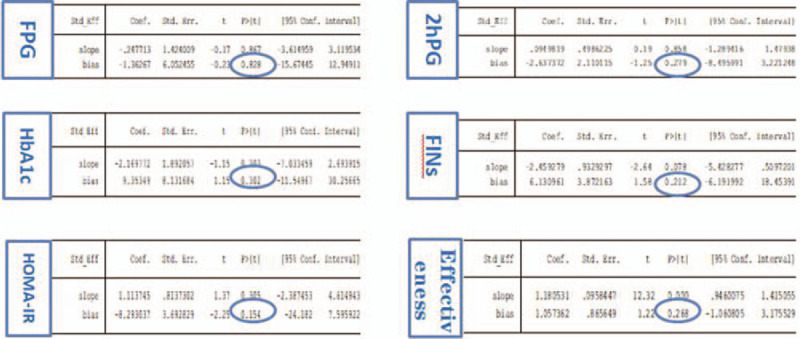
Data chart of bias analysis of Egger regression table.

In this study, we used “Egger” to analysis FPG, 2hPG, HbA1c, FINs, HOMA-IR, and the effective rate respectively by Stata, *P*-values were less than .05, no evident publication bias can be found. It suggests that the possibility of publication bias was small and the conclusion was reliable, as shown in Figure 20.

## 4 Discussion

T2DM is a kind of endocrine and metabolic disease with hyperglycemia as the main sign, which belongs to the category of “thirst quenching disease” in TCM. According to the theory of “strange diseases,” it tells that “People must be fat while eating too much sweet and fat.” People who are too fat must catch the syndrome of internal hot. Sweet in the stomach can be bloat. So Qi in such people will operate to the upper part, and then turns into thirst quenching. However, the obesity type T2DM with over eating fat and sweet is the main type of modern T2DM. It is found that about 80% of T2DM patients are obese or overweight. People who without “eat too much, drink too much and have too much urine” belongs to “Pi Dan.” “Pi Dan” should be diagnosed as actual situation, it can be treated with eliminating methods^[16]^. At present, western medicine is mainly used in clinical treatment, with certain curative effect and side effects, and the secondary failure will occur if taken for a long time.

The main mechanism of massage in the treatment of diabetes is to adjust the function of viscera, improve clinical symptoms, balance Yin and Yang, clear and activate the channels and collaterals, operate Qi and blood, nourish muscles and bones, and improve the function of viscera. From the perspective of modern medicine, massage can expand blood vessels, promote blood flow, improve microcirculation, promote insulin secretion,^[17]^ and improve the nervous centralis and the function of vegetative nervous system can enhance the immune function of the body, strengthen the metabolism of the body, make full use of the sugar in the muscle tissue, so as to achieve the purpose of reducing blood sugar and treating diabetes.^[18]^ It has the characteristics and advantages of multi-components, multi-channels, multi-targets, green, relatively safe, and small side effects.

The results of this meta-analysis shows that CM combined with metformin hydrochloride tablets in the treatment of T2MD has obvious advantages over metformin hydrochloride tablets alone in terms of Clinical Efficacy, FPG, 2hPG, HbA1c, FINs, TCMSSS, HOMA-IR, BMI index, and TC improvement. Ten studies^[6–15]^ all mentioned negative events. Metformin hydrochloride tablets can cause decreased appetite and nausea in small number of patients, when CM combined with metformin hydrochloride tablets only 3 studies mentioned that a small number of patients had decreased appetite and nausea. It is suggested that massage can be used as a clinical medicine reducing therapy for T2MD, which not only has a good effect, but also significantly reduces side effects and secondary failure. The heterogeneity of FPG, HbA1c, FINs, and TC data in this study may be related to drug dosage, treatment course, massage time, and operation method. The sensitivity analysis can be basically excluded.

In conclusion, it is feasible for CM to assist in the treatment of T2MD in adults. It can not only improve the clinical effect, but also improve the blood lipid, reduce the body weight to a certain extent, and it has less negative reactions. It provides reliable evidence for the feasibility of CM assisted treatment of adult T2MD patients, and it is a kind of recommended green clinical alternative therapy. The number of literatures included in this study is relatively small, and the quality of clinical evidence-based evidence is lower, which reduces the credibility of this study to a certain extent. We should be cautious about the results, so we need more high-quality, multi-centered, large sample, long follow-up, RCTs to verify. However, the ultimate goal of diabetes treatment is not only to make the blood glucose control normal or close to normal level for a long time, but also to fundamentally correct the metabolic disorder, prevent or delay the occurrence and development of various syndrome, actively explore the clinical curative effect scheme of reducing drug use without affecting the treatment effect of T2DM. It is worth further study by the experts in related fields. This research group will further follow-up the research.

## Author contributions

**Data curation:** Xiaolin Zhang, Di Cao.

**Formal analysis:** Xiaolin Zhang, Di Cao.

**Funding acquisition:** Mingjun Liu.

**Methodology:** Minghui Yan.

**Project administration:** Xiaolin Zhang, Mingjun Liu.

**Software:** Xiaolin Zhang, Di Cao.

**Supervision:** Mingjun Liu.

**Validation:** Mingjun Liu.

**Visualization:** Xiaolin Zhang, Di Cao.

**Writing – original draft:** Xiaolin Zhang, Mingjun Liu.

**Writing – review & editing:** Xiaolin Zhang, Di Cao.

## References

[1] Seuring T Archangelidi O Suhrcke M The economic costs of type 2 diabetes: a global systematic review. Pharmacoeconomics 2015;33:811–31. 25787932 10.1007/s40273-015-0268-9 PMC4519633

[2] Hu C Jia W Diabetes in China: epidemiology and genetic risk factors and their clinical utility in personalized medication. Diabetes 2018;67:3–11. 29263166 10.2337/dbi17-0013

[3] Mostafavinia A Amini A Ghorishi SKJ The effects of dosage and the routes of administrations of streptozotocin and alloxan on induction rate of type l diabetes mellitus and mortality rate in rats. Lab Anin Res 2016;32:160–5. 10.5625/lar.2016.32.3.160 PMC5057004 27729932

[4] GBD 2015 Disease and Injury Incidence and Prevalence Collaborators. Global regional, and national incidence, prevalence, and years lived with disability for 310 diseases and injuries, 1990-2015: asystematic analysis for the Global Burden of Disease Study 2015 [published correction appears in Lancet. 2017 Jan 7;389(10064):e1]. Lancet 2016;388:1545–602. 27733282 10.1016/S0140-6736(16)31678-6 PMC5055577

[5] Chinese Diabetes Society. Guidelines for the prevention and treatment of type 2 diabetes in China (2017). J Pract Med 2018;38:292–344.

[6] Xuesong L Jin B Juncai X Clinical study on the treatment of type 2 diabetes by spinal massage. Beijing Tradit Chin Med 2019;38:470–4.

[7] Wei Z Han Y Yunchuan W Clinical study on the intervention of ”transfer to Tongdu“ massage on the glucose metabolism of type 2 diabetes in community. New World Diabetes 2018;21:30–2.

[8] Renshan H Bijian Y Jinhui K To explore the clinical effect of traditional Chinese Massage on community type 2 diabetes. China Pract Med 2018;13:36–7.

[9] Xianbin WS Lianyan##Y Zhihong W Clinical study on the treatment of type 2 diabetes mellitus of phlegm dampness stagnation type by Tuina through regulating meridian. Clin J Acupunct 2017;33:36–8.

[10] Changchun University of Traditional Chinese Medicine, DeZhi Z Clinical Study on the Treatment of Dyslipidemia in Type 2 Diabetes Mellitus (Deficiency of Both Qi and Yin) by Massage Method of ”Transporting Abdomen and Dredging Channels“. 2016.

[11] Changchun University of Traditional Chinese Medicine, Han Yuxi Clinical Study on the Treatment of Type 2 Diabetes Mellitus with Deficiency of Both Qi and Yin by ”Yunfu Tongjing Method". 2015.

[12] Zhaohui W Zhihong W Bolin S Effect of abdominal massage combined with metformin on glucose and lipid metabolism in obese type 2 diabetic patients. Chin J Gerontol 2014;34:6874–5.

[13] Jun Z dujun M Yuhang W Clinical observation and study of massage manipulation in the treatment of early type 2 diabetes. China Med J 2013;10:115–7.

[14] Bailin S Chunli P Zhihong W Clinical observation on 80 obese patients with type 2 diabetes treated by massage and metformin. World J Integr Tradit Chin West Med 2011;6:206–9.

[15] Shiqin Z Clinical observation on 24 cases of type 2 diabetes treated with massage and drugs. Jilin Tradit Chin Med 2008;177.

[16] Xiaolin T Review of TCM cognition and research progress on diabetes mellitus. Beijing J Tradit Chin Med 2016;35:509–12.

[17] Wändell PE Ärnlöv J Nixon Andreasson A Effects of tactile massage on metabolic biomarkers in patients with type 2 diabetes. Diabetes Metab 2013;39:411–7. 23642641 10.1016/j.diabet.2013.02.002

[18] Wändell Per E Carlsson Axel C Catharina G Measuring possible effect on health-related quality of life by tactile massage or relaxation in patients with type 2 diabetes. Complement Ther Med 2012;20:8–15. 22305243 10.1016/j.ctim.2011.09.007

